# Outcome of catheter ablation of supraventricular tachyarrhythmias in cardiac sarcoidosis

**DOI:** 10.1002/clc.23263

**Published:** 2019-09-03

**Authors:** Kevin Willy, Dirk G. Dechering, Kristina Wasmer, Julia Köbe, Nils Bögeholz, Christian Ellermann, Patrick Leitz, Florian Reinke, Gerrit Frommeyer, Lars Eckardt

**Affiliations:** ^1^ Department for Cardiology II: Electrophysiology University Hospital Münster Münster Germany

**Keywords:** cardiac sarcoidosis, catheter ablation, inflammatory heart disease, sarcoidosis

## Abstract

**Background:**

Sarcoidosis is a multisystem granulomatous disease of not sufficiently understood origin. Some patients develop cardiac involvement in course of the disease which is mostly responsible for adverse outcome. In addition to complications like high degree atrioventricular (AV) block or ventricular tachyarrhythmias, there is a certain percentage of patients developing atrial tachyarrhythmias. Data is limited and the role of catheter ablation uncertain. Therefore, we studied sarcoid patients who presented with supraventricular tachyarrhythmias.

**Hypothesis:**

Treatment and ablation of supraventricular tachycardia could be hampered by inflammation in patients with cardiac sarcoidosis.

**Methods:**

We enrolled 37 consecutive patients with cardiac sarcoidosis who presented with atrial tachyarrhythmias and underwent an electrophysiologic study over a period of 6 years (03/2013‐04/2019). In total, 16 catheter ablations for atrial tachyarrhythmias were performed. Mean follow‐up duration was 2.5 years.

**Results:**

Most common ablation performed was cavo‐tricuspid isthmus ablation for typical atrial flutter in seven patients (54%). Pulmonary vein isolation for treatment of atrial fibrillation (AF) was performed in five patients (38%). Two patients received slow‐pathway modulation for treatment of recurrent atrioventricular nodal reentry tachycardia (AVNRT). All but two patients with AF had no clinical recurrence during follow‐up. Two patients had recurrence of AF but still reported markedly improved european heart rhythm association (EHRA) class. Periprocedural safety was very high. There were no adverse events related to the ablation procedure. One patient died during follow‐up in the presence of electrical storm.

**Conclusion:**

Catheter ablations of supraventricular tachycardias seem to be safe and effective in patients with cardiac sarcoidosis. Outcome is comparable to patients without inflammatory heart disease, although data from larger patient collectives are mandatory to make recommendations in this special entity.

## 1 INTRODUCTION

Sarcoidosis is a chronic multisystem granulomatous disease of uncertain etiology and with relevant geographical and ethnical differences.^1^, ^2^ The most severe form for the majority of patients is cardiac involvement. Cardiac sarcoidosis (CS) can manifest itself with multiple cardiac arrhythmias, of which high degree AV‐block and ventricular tachycardias are the most dangerous complications. Supraventricular tachycardias are less common or at least less well described. According to previous studies there is a mentionable amount of CS patients who suffer from atrial arrhythmia.^3^, ^4^, ^5^, ^6^ In a cohort of 100 CS patients one third of patients developed atrial tachyarrhythmias during a relatively short follow up of 21 months.^7^ Underlying pathophysiology includes sarcoid‐dependent inflammation, scarring, and electrophysiological heterogeneity of the atria as well as atrial remodeling due to elevated atrial pressure in case of impaired ventricular ejection fractions or pulmonary hypertension.^8^

As catheter ablation has evolved as the gold standard for the majority of supraventricular tachycardias, the question whether the very favorable outcome of such procedures is also valid for patients with cardiac sarcoidosis, is of clinical importance. As the underlying mechanism of arrhythmias in the general population is thought to be different from patients with inflammatory heart disease such as sarcoidosis one may speculate that sarcoid patients have worse outcome.^9^, ^10^ Data on ablation procedures of supraventricular tachycardias in these patients with sarcoidosis are sparse, so that to our knowledge, the present study represents the largest data set concerning catheter ablation of supraventricular tachyarrhythmias in patients with cardiac sarcoidosis.

## 2 METHODS

The study was conducted in accordance with the guidelines of the Declaration of Helsinki. In the present single‐center retrospective study we identified 37 patients with sarcoidosis who received one or more electrophysiogical study.

Cardiac manifestation of the sarcoidosis was diagnosed by either typical late gadolinium enhancement (LGE) in cardiac magnetic resonance imaging (MRI) or by biopsy.

While 12 patients underwent the electrophysiologic study for risk stratification and 12 patients underwent ventricular tachycardia (VT) ablation, 13 turned out to have an electrophysiologic study performed for documented supraventricular tachycardias. All procedures were performed in the University Hospital of Münster, Germany, between March 2013 and April 2019.

### 2.1 Ablation procedure

Five patients with atrial fibrillation (AF) underwent pulmonary vein isolation (PVI) with the cryo‐balloon technique using the Medtronic ArcticFront Cryoballon 12F catheter (Medtronic, Dublin, Ireland). For re‐do procedures (1 patient) we chose radio frequency (RF)‐ablation with 3D‐ mapping (NavX; Abbott, St. Paul, Minnesota). Ablations of the cavo‐tricuspid isthmus (CTI) (n = 7) for atrial flutter (AFL) were performed with RF ablation using an 8 mm‐tip catheter (Biotronik AlCath Black full circle; Biotronik, Berlin, Germany). In case of AVNRT (n = 2), slow pathway modulation was performed using RF ablation with a 4 mm‐tip, catheter (Osypka Cerablate easy; Osypka AG, Rheinfelden, Germany). Surface electrocardiograms (electrocardiograms (ECGs) and bipolar and unipolar intracardiac electrograms were registered in a digital recording system. Signals were sampled at 1 kHz and filtered at 0.1‐100 Hz for surface ECGs and at 30‐250 Hz for intracardiac signals according to our clinic protocol.^11^

### 2.2 Follow‐up

Mean duration of follow‐up was 2.5 ± 2.4 years (median follow‐up 2 years). Duration between one to 2766 days. Follow‐up was recorded if the patient was seen in our outpatient clinic or if they were hospitalized in our clinic.

## 3 RESULTS

Out of 37 consecutive patients with an established diagnosis of CS, who underwent electrophysiological examination in our clinic, 13 (35%) had supraventricular tachycardias as the indication for the electrophysiological examination. In these 13 patients, 16 ablation procedures were performed in total. Seven of these 13 patients (53%) required catheter ablation for atrial flutter. In one patient re‐ablation was performed due to recovery of the cavotricuspid isthmus. Two patients underwent slow‐pathway modulation for AVNRT. Five patients received catheter ablation for AF (38%, PVI). One patient with AF also was ablated for atrial flutter. Another patient underwent two PVI procedures because of recurrent AF with proven recovery of the left upper pulmonary vein. Another patient received a PVI in another hospital 2 years before and was referred to our clinic for a re do procedure in which re‐ablation of the two upper pulmonary veins was performed.

Table 1 shows patient characteristics at the time of ablation as well as information about outcome during follow‐up. Patients were 45 to 76 years (mean age 58.4 ± 8.9 years) and 9 of 13 patients were male (69%). All patients received beta‐blockers, and seven of 13 patients (53%) received a trial of antiarrhythmic drug therapy prior to ablation. Of note, seven of 13 (53%) patients received immunosuppressive therapy for active sarcoidosis. Five of 13 patients had evidence of left ventricular (LV) systolic dysfunction (overall mean LV‐ejection fraction (EF) 54.5 ± 8.9%). Six of 13 patients had a cardiac device implanted (3 implantable cardioverter defibrillator (ICD), [two after monomorphic VT, one for primary prophylaxis], 3 pacemaker (PMs) [two for high degree AV block, one for bradyarrhythmic atrial fibrillation]). One patient had multiple ablations for recurrent VT and finally died due to septic pneumonia during follow‐up.

**Table 1:** Patient characteristics and outcome data

Patient	Type of ablation	ECG	Medication	Medical history/outcome
Pat 1, M, 45 yo, NYHA III	CTI ablation	Right bundle branch block (RBBB), left anterior hemiblock (LAHB)	Amiodarone; Azathioprine+ Prednisolone	Death (pneumogenic sepsis), Several episodes of electrical storm with shock delivery of cardiac resynchronisation therapy (CRT)‐ICD, 3 VT ablation procedures
Pat 2, M, 55 yo, NYHA II	CTI ablation, CTI re‐ablation, PVI with cryoballoon		Amiodarone/Prednisolone	Recurrent typical AFL due to recovery of the CTI 3.5 years after the first ablation
Pat 3, M, 52 yo, NYHA I	PVI with cryoballoon	AV I°, LAHB	No specific	No recurrency during follow‐up
Pat 4, M, 47 yo, NYHA III	CTI ablation		Methotrexat (MTX) + Prednisolone	Several exacerbations of pulmonary sarcoidosis during follow‐up, development of oligosymptomatic (EHRA IIa) AF
Pat 5, F, 69 yo, NYHA I	Slow‐Pathway‐Modulation	AV I°, LAHB	MTX + Prednisolone	No recurrency of AVNRT. Development of complete left bundle branch block (LBBB), worsening of LV‐EF to 40% and NYHA III during follow‐up
Pat 6, M, 62 yo, NYHA II	CTI ablation	AV I°, LAHB	None	Development of AV III° with consecutive dual dual dual (DDD)‐PM implantation
Pat 7, M, 47 yo, NYHA II	PVI with cryoballon, Re‐isolation of LSPV with RF		Azathioprine + Prednisolone	2 oligosymptomatic, self‐limited AF episodes after 2nd PVI, watchful waiting
Pat 8, F, 66 yo, NYHA III	Re‐isolation of right superior pulmonary vein (RSPV) and left inferior pulmonary vein (LIPV) after PVI 2 years ago in another hospital		None	DDD‐PM for bradyarrhythmia absoluta, upgrade to CRT due to high ventricular stimulation rate
Pat 9, M, 64 yo, NYHA II	CTI ablation	LAHB	None	Sinus arrest with 15 seconds pause ➔ DDD‐PM implantation
Pat 10, M, 66 yo, NYHA II	PVI with cryoballoon		Dronedarone	Asymptomatic recurrence, rate control
Pat 11, M, 76 yo, NYHA II	CTI ablation	AV I°, incomplete RBBB	Mycophenolat+ Prednisolone	Also renal involvement of sarcoidosis
Pat 12, F, 62 yo, NYHA I	CTI ablation			No recurrence during follow‐up
Pat 13, F, 56 yo, NYHA I	Slow‐Pathway‐Modulation	LAHB	MTX + Prednisolone	Ventricular ventricular Inhibition (VVI)‐ICD implantation due to sustained monomorphic VT

Abbreviations: AF, atrial fibrillation; AFL, atrial flutter; ECG, electrocardiogram.

Two patients had recurrence of AF during follow‐up. One patient with recurrent AF was asymptomatic (EHRA I), while symptomatology was severe before PVI (EHRA III). The other patient had only two very short symptomatic recurrences of AF, which he could terminate reliably with flecainide, so that anti‐arrhythmic therapy was continued on the basis of a “pill‐in‐the‐pocket” concept.

## 4 DISCUSSION

While supraventricular tachycardias are a common finding in CS, they are not an immediately dangerous condition in most cases, but patients are frequently symptomatic despite medical therapy. Even worse, in structural heart diseases like CS some patients tend to suffer from heart failure symptoms or may even develop a tachycardiomyopathy. These patients require catheter ablation not only to restore sinus rhythm for symptom relief, but also for preserving left ventricular function. Furthermore, options for antiarrhythmic drug therapy are limited due to the structural cardiac abnormalities induced by the (ongoing) inflammation as well as the presence of AV conduction disorders.

Patients in our study were relatively young with a similar mean age compared to the cohort in the study of Willner et al^7^ Surprisingly, 9 of the 13 patients were male while typically sarcoidosis patients are predominantly middle‐aged women.^12^ While older studies have shown no gender difference in cardiac sarcoidosis,^2^, ^13^, ^14^ more recent trials from Germany^15^ and Poland^16^ found cardiac involvement to be more common in male sarcoidosis patients. In a retrospective analysis of over 1300 sarcoidosis patients, cardiac involvement was proven in 64 patients in this analysis, of whom 70% were men.^16^ These data indicate that cardiac manifestation may be gender‐specific.

Concerning drug treatment, over 50% of the patients received immunosuppressive agents due to an active sarcoidosis reflecting the sick patient collective and the fact that treatment of arrhythmia could be difficult to handle successfully during ongoing inflammatory processes. Likewise, over half of patients received insufficiently effective antiarrhythmic drug therapy prior to undergoing an ablation procedure. Furthermore, 3 of 13 patients had an ICD, 2 of them due to history of sustained ventricular arrhythmia and one patient even suffered from electrical storm and was subject to VT ablations.

### 4.1 Role of catheter ablation of supraventricular tachycardias in cardiac sarcoidosis

According to previous studies, the pathophysiology of supraventricular tachyarrhythmias in CS is multifactorial.^5^, ^7^ Cavo‐tricuspid isthmus dependent AFL may be related to pulmonary hypertension and adverse right atrial remodeling due to elevated right ventricular pressure. This situation is worsened by the inflammatory nature of sarcoidosis and the resulting development of myocardial scars and conduction delays. While Willner et al^7^ reported that atrial scars and sarcoid granulomas seemed to be more common in the left atrium in CS patients, in our group cavo‐tricuspid AFL was the most common arrhythmia leading to an indication for catheter ablation. As typical AFL is also a very common reason for catheter ablation in the absence of cardiac sarcoidosis, it remains unclear whether granuloma infiltration plays a key role in the development of typical AFL in these patients. Of note the mean atrial cycle length of AFL in our patient cohort seemed comparable to patients without cardiac sarcoidosis as was the mean conduction time via the isthmus after ablation induced block of 160 ms from the CS ostium to the inferior lateral atrium and vice versa. Regardless of the underlying substrate, the results of the present study suggest that catheter ablation of supraventricular tachyarrhythmias, even in case of pulmonary vein isolation for atrial fibrillation, has a high immediate and short‐to‐mid‐term procedural success rate. Although recurrences may be difficult to avoid due to the often progression of the disease itself and despite the limited number of patients, these results are promising and may encourage clinicians to perform catheter ablation of supraventricular tachycardias in cardiac sarcoidosis. In our study, catheter ablations such as PVI could prevent symptomatic recurrence of AF (3 patients) or at least reduce the symptom burden, so that no additional ablation was necessary (2 patients). CTI ablations had an even higher succession rate as expected. Only one patient had a recurrence of typical flutter due to conduction recovery of the cavo‐tricuspid isthmus. Thus, ablation was safe and effective in our small patient cohort independently of the diffuse character of sarcoidosis itself. Besides, we may speculate that arrhythmia development seems to be at least partially similar to patients without inflammatory cardiac disease because, as stated above, CTI ablation as well as PVI were comparably effective in CS patients.

### 4.2 Study limitations

This is a retrospective, single‐center study with a limited number of patients due to the rare entity of cardiac sarcoidosis. Furthermore, the trial was not designed to identify underlying pathophysiology for the development of supraventricular tachycardias in sarcoidosis but shall report the results of catheter ablations in such cases. Follow‐up duration was intermediate to long‐term with about 3 years of duration, but very long‐term outcomes concerning recurrence and disease progression were not available. Additionally, patients with supraventricular tachycardias not considered for an electrophysiologic study and catheter ablation were not recorded, so that the distribution of ablation procedures might be biased.

## 5 CONCLUSION

In patients with cardiac sarcoidosis, supraventricular tachycardias are not as good characterized as ventricular tachycardia or AV block but still a certain number of patients suffer from these arrhythmias. Patients with cardiac sarcoidosis are often young at the time of diagnosis and are therefore no ideal candidates for a long‐term treatment with antiarrhythmic drugs, especially with amiodarone and may not be candidates for Class I antiarrhythmic drugs. Typical AFL was the most common arrhythmia followed by AF in our patient collective. Catheter ablation could be performed safely and with high success rates. Only two re‐do procedures had to be performed due to arrhythmia recurrences. In all patients, the ablation procedure could at least ease the symptomatology or prevent symptomatic recurrence at all. Further prospective studies have to define the definitive role of ablation for supraventricular tachycardias in cardiac sarcoidosis.
